# Blebbistatin as a novel antiviral agent targeting equid herpesvirus type 8

**DOI:** 10.3389/fvets.2024.1390304

**Published:** 2024-06-05

**Authors:** Liangliang Li, Xiu Cui, Yue Yu, Qi Sun, Wenjing Li, Yubao Li, Shuwen Li, Li Chen, Muhammad Zahoor Khan, Changfa Wang, Tongtong Wang

**Affiliations:** College of Agronomy, Liaocheng University, Liaocheng, China

**Keywords:** EqHV-8, blebbistatin, myosin II ATPase inhibitor, antiviral activity, animal model

## Abstract

**Introduction:**

Equid herpesvirus type 8 (EqHV-8) poses a significant threat to equine health, leading to miscarriages and respiratory diseases in horses and donkeys, and results in substantial economic losses in the donkey industry. Currently, there are no effective drugs or vaccines available for EqHV-8 infection control.

**Methods:**

In this study, we investigated the *in vitro* and *in vivo* antiviral efficacy of Blebbistatin, a myosin II ATPase inhibitor, against EqHV-8.

**Results:**

Our results demonstrated that Blebbistatin significantly inhibited EqHV-8 infection in Rabbit kidney (RK-13) and Madin-Darby Bovine Kidney (MDBK) cells in a concentration-dependent manner. Notably, Blebbistatin was found to disrupt EqHV-8 infection at the entry stage by modulating myosin II ATPase activity. Moreover, *in vivo* experiments revealed that Blebbistatin effectively reduced EqHV-8 replication and mitigated lung pathology in a mouse model.

**Conclusion:**

Collectively, these findings suggest that Blebbistatin holds considerable potential as an antiviral agent for the control of EqHV-8 infection, presenting a novel approach to addressing this veterinary challenge.

## 1 Introduction

Equid herpesvirus type 8 (EqHV-8), also known as Asinine Herpesvirus 3 (AHV-3), is a pathogen that causes severe respiratory disease and miscarriages in equines, posing a persistent threat to horse and donkey farming worldwide (1, 2). The EqHV-8 belongs to the Alpha Herpesviridae family, It is characterized as a double-stranded DNA virus with a genome length of approximately 150 kilobases and comprising 76 open reading frames (3). In recent years, EqHV-8 has shown a significant increase in prevalence, particularly in large-scale donkey farms in China. For instance, Wang et al. conducted a study indicating an EqHV-8 infection rate of approximately 38.7% (457/1180) in donkey farms in Shandong province, China (4). Additionally, cases of abortion and neurological diseases in donkeys induced by EqHV-8 infections have been documented (5, 6), highlighting the potential threat to the donkey industry. While the molecular mechanisms underlying EqHV-8 infections have been well documented in previous studies (7–10), it is crucial to note the limited availability of effective drugs for combating EqHV-8 infections (9, 10).

Blebbistatin, known for its inhibitory properties on myosin II ATPase activity, has gained significant attention and utility in biochemical, cell biological, and physiological research (11, 12). Recent studies have also explored its antiviral properties against various viruses. Notably, Antoine et al. demonstrated Blebbistatin’s ability to significantly reduce the entry of Herpes Simplex Virus Type-1 (HSV-1) into human corneal epithelial (HCE) cells by interfering with myosin light chain kinase (13). In a study by Gao et al. (14), Blebbistatin exhibited inhibitory effects on Porcine reproductive and respiratory syndrome virus (PRRSV) infection. This inhibitory effect was observed in multiple contexts, including *in vitro* experiments using MARC-145 cells and PAM cells, as well as in swine (14). Moreover, Li et al. highlighted Blebbistatin’s potential to mitigate PRRSV infection in other susceptible cell lines, such as PK-15^CD163^, HEK-293T^CD163^, and BHK-21^CD163^ (15). Additionally, Blebbistatin has demonstrated the capacity to suppress the replication of Murine Gammaherpesvirus 68 (MHV-68) both *in vitro* and *in vivo* (16). Despite the growing body of evidence regarding Blebbistatin’s antiviral properties, its effectiveness against EqHV-8 remains unexplored.

In this study, we aimed to investigate the anti-EqHV-8 activity of Blebbistatin and elucidate its underlying mechanism. Our results demonstrate that Blebbistatin significantly reduces EqHV-8 replication in Rabbit kidney (RK-13) and Madin-Darby Bovine Kidney (MDBK) cells. Furthermore, Blebbistatin exerts its inhibitory effects at the adsorption and internalization stages of EqHV-8. Notably, Blebbistatin also displayed efficacy in decreasing EqHV-8 replication in lung tissue within a mouse model. In summary, our research highlights Blebbistatin as a promising candidate for controlling EqHV-8 infections.

## 2 Materials and methods

### 2.1 Cells, EHV-8 strain and reagents

The RK-13 and MDBK cells were cultured in Modified Eagle’s medium (MEM, Gibco, United States) supplemented with 10% fetal bovine serum (FBS, Gibco, United States) and 1% penicillin–streptomycin (Servicebio, Wuhan, China) at 37°C and 5% CO_2_. The EHV-8 SDLC66 strain (GenBank: MW816102.1), SD2020113 (GenBank: MW822570.1), and donkey/Shandong/10/2021 (GenBank: OL856098.1) were propagated in RK-13 cells. Additionally, Blebbistatin was obtained from Shandong Sikejie Biotechnology Co., LTD (Jinan, China) and dissolved in dimethyl sulfoxide (DMSO) (Solarbio, Beijing, China).

### 2.2 Cell viability detection

The cytotoxicity of Blebbistatin was detected using the Cell Counting Kit-8 (CCK-8) assay (Beyotime, Nanjing, China) as previously described (17). Briefly, RK-13 or MDBK cells were seeded into a 96-well plate at a density of 1 × 10^4 cells per well and incu-bated with Blebbistatin at various concentrations (0, 2.5, 5, 10, 20, 40, and 80 μM) for 24 h. Subsequently, the CCK-8 reagent was added to each well according to the manufacturer’s instructions (10 μL/well) and incubated at 37°C for 2 h. The viability of the cells was determined by measuring the absorbance at 450 nm and analyzed us-ing the formula “cell survival rate (%) = [OD (sample) - OD (blank)/OD (control) -OD (blank)] × 100%.”

### 2.3 DNA/RNA extraction and qPCR analysis

The DNA supernatant virus from different samples was extracted using the DNA Viral Genome Extraction Kit (Solarbio, Beijing, China). Absolute quantification PCR (qPCR) assay was performed to detect the supernatant DNA copies of EqHV-8 with recombinant plasmids pMD18-T-gD and *ORF72*-F and *ORF72*-R primers, as previously established method (10), and calculated by normalization to the standard curve.

The isolation of total RNA from cellular samples was accomplished employing TRIzol reagent (Sparkjade, Jian, China), following established procedures (10). Subsequently, the RNA was reversed to complementary DNA (cDNA) by All-in-One First Strand cDNA Synthesis Kit (GENENODE, Wuhan, China). The ensuing reactions were conducted on a Step One Plus real-time PCR system, wherein the SYBR™ Green PCR Master Mix (Genstar, Beijing, China) was employed in conjunction with specific primers designed to target the gD gene of EqHV-8. For the purpose of normalizing the gene expression data, mRNA expression levels of Glyceraldehyde-3-Phosphate Dehydrogenase (*GAPDH*) were utilized as an internal reference, in accordance with well-established practices (14). The quantification of the target gene expression was carried out employing the 2^-ΔΔCt^ method, a widely accepted methodology previously described in the literature (14). The primer sequences are listed in Supplementary Table S1.

### 2.4 Western blot assay

The western blot assay was conducted in accordance with established protocols (18). In brief, cellular samples were collected at designated time intervals and subsequently lysed using NP40 lysis buffer. The lysates were mixed with 5 × protein loading buffer and separated via 12% SDS-PAGE gels. Following gel separation, the proteins were transferred onto polyvinylidene difluoride (PVDF) membranes. These membranes were subjected to blocking with 5% nonfat dry milk for a duration of 1 h. Subsequently, they were incubated with primary antibodies, specifically mouse anti-EqHV-8 gD, or mouse anti-α-Tubulin antibodies, and washed using PBST. Following the primary antibody incubation, the membranes were treated with a secondary antibody, the HRP-conjugated goat anti-mouse antibody. Ultimately, the visualization and imaging of specific protein bands were accomplished utilizing the ChemiDoc MP Imaging System (BioRad, California, United States).

### 2.5 Virus titration

The quantification of viral progeny production was executed through a titration procedure as previously documented (6). In brief, RK-13 cells were cultured in 96-well plates at a density of 1×10^4 cells per well and allowed to incubate overnight. The viral supernatant was serially diluted by a factor of 10, with each dilution replicated eight times and added at a volume of 100 μL per well. These cells were then incubated at 37°C for a duration of 3–5 days, during which the cytopathic effect (CPE) was observed daily. The 50% cell culture infectious dose (TCID_50_) was subsequently calculated employing the Reed–Muench method.

The virus titers in lung samples of EqHV-8-infected mice were detected using RK-13 cells. Briefly, all mice from 3 groups were sacrificed at 7 dpi, and their lungs were collected. The lung tissues (0.1 g) were mixed with PBS (1 mL), further crushed, homogenized, frozen and thawed 3 times. The supernatant was obtained from lung tissues by centrifugation, and filtered to remove bacteria using 0.22 μm syringe filter. Finally, the virus titer was determined in RK-13 cells as above.

### 2.6 Antiviral activity analysis

RK-13 and MDBK cells were seeded in 12-well plates and pre-treated with various concentrations of Blebbistatin (0, 2.5, 5, 10, and 20 μM) for 1 h. Subsequently, they were infected with EqHV-8 SDLC66 at a multiplicity of infection (MOI) of 0.1 for 1 h. Following infection, the cells were cultured in 3% FBS MEM containing Blebbistatin at the indicated concentrations. Cells were collected at 24-h post infection (hpi) to assess EqHV-8 replication via western blot analysis. Additionally, the cellular supernatants were also harvested for the detection of progeny virus titer using TCID_50_.

### 2.7 Indirect immunofluorescence assay

To assess the antiviral efficacy against various EqHV-8 strains, an IFA was employed. In brief, RK-13 cells were pretreated with Blebbistatin at concentrations of 0, 5, 10, and 20 μM for a duration of 1 h. Subsequently, these cells were infected with EqHV-8 strains, including SDLC66, SD2020113, or Donkey/Shandong/10/2021, each at0.1 MOI for 1 h. Following infection, the cells were washed with PBS and then incubated in Modified Eagle Medium (MEM) containing 3% fetal bovine serum (FBS) and the indicated dosage of Blebbistatin. At 36 hpi, these cells were fixed with paraformaldehyde, permeabilized with Triton X-100, and subjected to washing with PBS. These cells were subsequently treated with mouse anti-EqHV-8-positive serum (produced in-house) and incubated with a secondary Rhodamine-conjugated goat anti-mouse IgG antibody. Finally, the cells were counterstained with 4′,6-diamidino-2-phenylindole (DAPI) and visualized using a DMi8 microsystems (Leica, Germany).

### 2.8 Analysis of temporal dynamics in EqHV-8 life cycle perturbation by Blebbistatin

To elucidate the specific phase of the EqHV-8 life cycle affected by Blebbistatin, RK-13 cells were cultured in 12-well plates under varying treatment conditions. These included distinct stages of exposure to EqHV-8 (MOI = 0.1) and 20 μM Blebbistatin, such as pre-treatment, co-treatment, post-treatment, and exposure throughout all stages. Subsequent to these treatments, the cells were harvested for the assessment of viral replication, utilizing quantitative PCR (qPCR) and western blot analyses at 24 hpi.

### 2.9 Assays for virus adsorption and internalization

In the virus adsorption assay, RK-13 cells, pre-seeded in 12-well plates, were treated with either 20 μM Blebbistatin or DMSO for 1 h at 37°C. Following treatment, the cells were cooled on ice and exposed to EqHV-8 SDLC66 (MOI = 1) for 1 h. Then, cells were washed thrice with pre-chilled PBS to remove non-adherent virions. The *mRNA* expression levels of bound EqHV-8 particles were quantified via qPCR.

For the virus internalization assay, RK-13 cells in 12-well plates were pre-treated with 20 μM Blebbistatin or DMSO at 37°C for 1 h, followed by incubation with EqHV-8 SDLC66 (MOI = 1) at 4°C for 1 h to facilitate virus binding. Unbound viruses were removed with chilled PBS, and the cells were incubated in 3% FBS MEM containing either Blebbistatin or DMSO at 37°C for 1 h to initiate internalization. Post-internalization, non-internalized viruses were eliminated using citrate buffer (pH 3.0), and the mRNA levels of EqHV-8 gD expression were detected by qPCR.

### 2.10 *In vivo* anti-EqHV-8 efficacy assay

Fifteen SPF (Specific Pathogen-Free) male BALB/c mice, aged 6 weeks, were procured from Pengyue (Jinan, Shandong Province, China), and were assigned randomly into three distinct experimental groups (n = 5/group). The first group, termed the ‘mock group,’ received intranasal administration of MEM in a volume of 50 μL (including 0.1% Dimethyl Sulfoxide, DMSO). The second group, designated as the ‘EqHV-8 group,’ underwent a pre-inoculation process in which they received intranasal administration of a solution consisting of 0.1% DMSO in MEM (50 μL). Subsequently, they were subjected to EqHV-8 inoculation at a viral concentration of 1 × 10^5 PFU (Plaque-Forming Units) per mouse. The third group, denoted as the ‘Blebbistatin+EqHV-8 group’. These mice were pre-inoculated intranasally with Blebbistatin at a dosage of 30 μM/kg in MEM (50 μL) at three specific time points: 1 day pre-infection, 1-day post-infection (dpi), and 3 dpi. Following this, they were subjected to EqHV-8 inoculation at the same viral concentration of 1 × 10^5 PFU per mouse. All inoculations were performed under profound anesthesia induced using Zoletil 50(Virbac in Nice, France). Throughout the duration of the study, the mice were provided with *ad libitum* access to food and water, and were individually housed to prevent potential cross-contamination. Clinical symptoms were meticulously monitored throughout the experimental period. On the 8th dpi, humane euthanasia was conducted via cervical dislocation, and lung tissues were harvested for subsequent histopathological analysis and evaluation of viral replication efficiency.

### 2.11 Histopathological evaluation

The protective effect of Blebbistatin against lung damage induced by EqHV-8 was evaluated through hematoxylin and eosin (H&E) staining methodology, as outlined in previous study (7). Lung tissues were meticulously preserved by immersing them in a 10% formalin solution. Following fixation, the tissues were embedded in paraffin wax, and subsequently, thin sections measuring 4 μm in thickness were meticulously prepared. These sections were then carefully mounted onto glass slides and subjected to the H&E staining procedure. The stained tissues were subsequently examined using light microscopy.

### 2.12 Statistical analysis

Data were processed and analyzed using GraphPad Prism 8.0 (San Diego, CA, United States). Differences between groups were assessed using the unpaired Student’s t-test. Statistical significance was denoted as follows: *, *p* < 0.05; **, *p* < 0.01; ***, *p* < 0.001.

## 3 Results

### 3.1 Assessment of Blebbistatin’s impact on cellular viability

**Figure 1:**
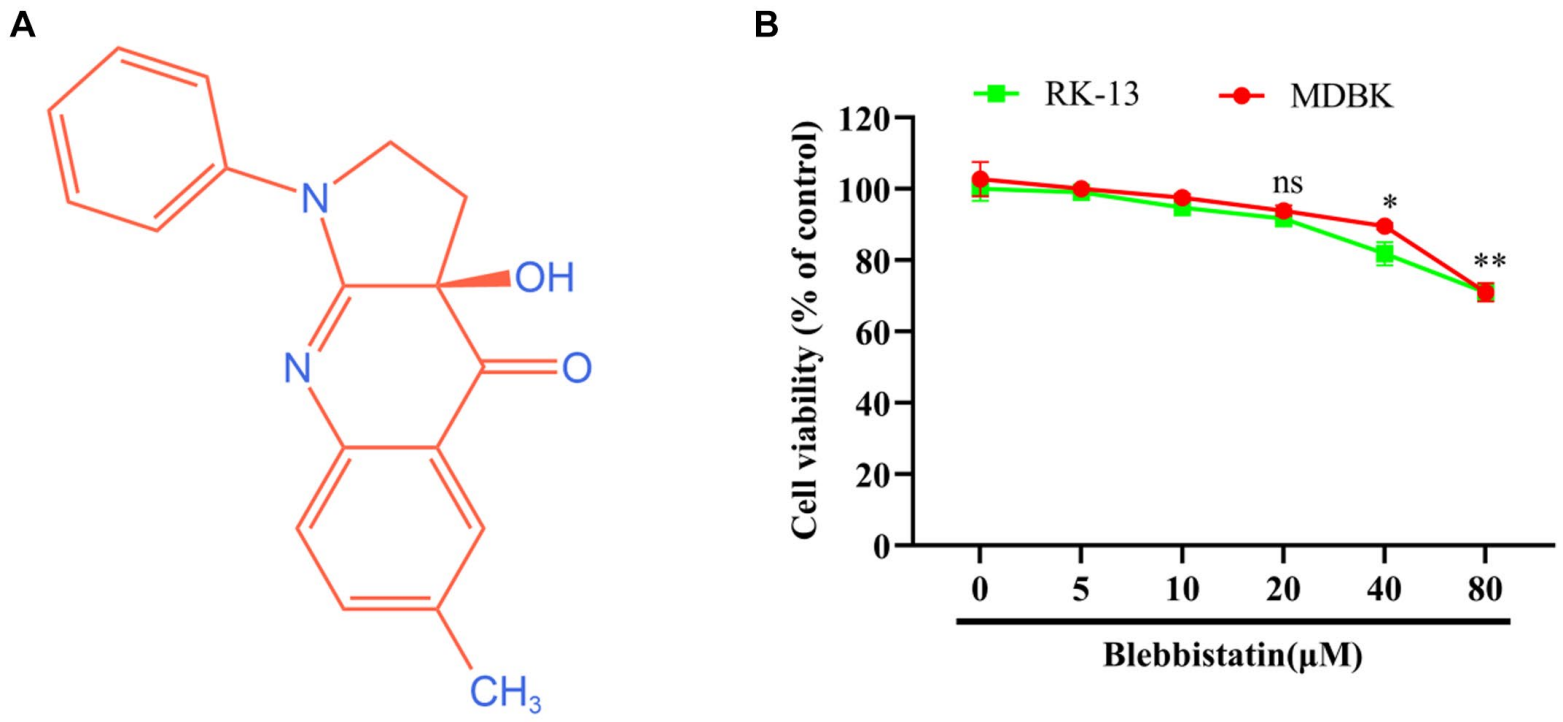
The chemical structure and cytotoxicity of Blebbistatin. **(A)** The chemical structure of Blebbistatin. **(B)** The cytotoxicity of Blebbistatin at different concentrations was determined in RK-13 and MDBK cells by CCK-8 kit and was expressed as relative cell viability by comparing with the viable cells in the absence of Blebbistatin (set up as 100%). These data shown are representatives from three independent experiments. *p < 0.05; **p < 0.01, ns: no significant.

The chemical structure of Blebbistatin is represented in Figure 1A. To assess the potential cytotoxic effects attributed to Blebbistatin, we conducted experiments involving RK-13 and MDBK cells. These cells were exposed to different concentrations of Blebbistatin, and subsequently, the CCK-8 reagent was introduced. Our findings revealed that the concentrations of Blebbistatin up to 20 μM exhibited no discernible impact on cell viability (*p* > 0.05) in either MDBK or RK-13 cell lines, as depicted in Figure 1B.

### 3.2 Inhibition of EqHV-8 infection by Blebbistatin in susceptible cells

**Figure 2:**
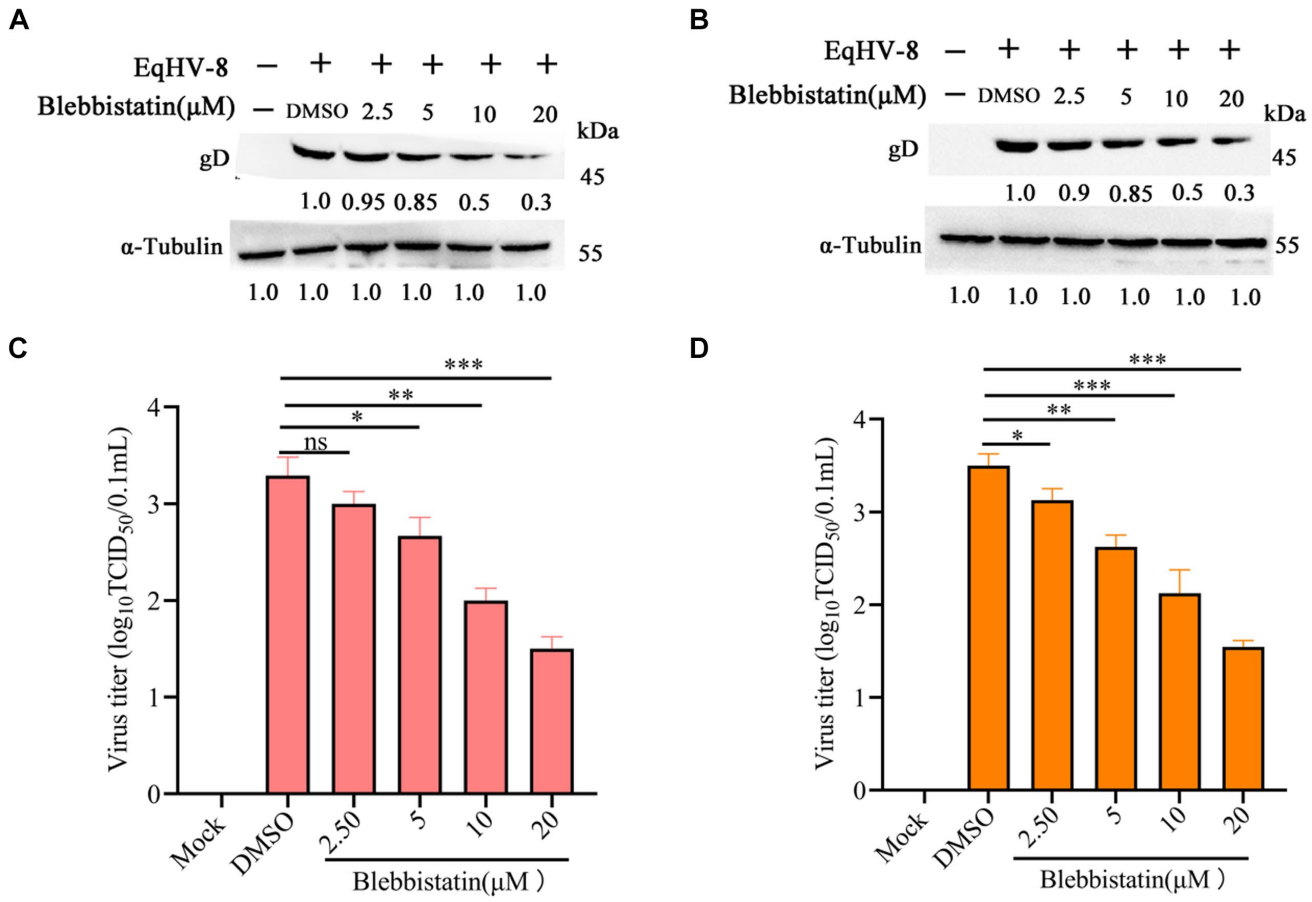
Blebbistatin inhibited EqHV-8 replication in susceptible cell. RK-13 **(A,B)** or MDBK **(C,D)** cells were pretreated with Blebbistatin at different concentrations (0, 2.5, 5, 10, and 20 μM) for 1 h, then infected with EqHV-8 SDLC66 (0.1 MOI) for 1 h at 37°C, then these cells were collected to detect gD expression by western blot at 24 hpi. Meanwhile, those cellular supernatants were harvested to assess virus titer using TCID_50._ Data were presented as the means of normalized data ± standard deviations (error bars) based on at least three independent experiments. * p < 0.05; ** p < 0.01; *** *p* < 0.001, ns: no significant.

To assess the potential anti-EqHV-8 effect of Blebbistatin, the RK-13 and MDBK cells were subjected to pretreatment with Blebbistatin at different concentrations for 1 h. Following this pre-incubation period, these cells were exposed to EqHV-8 SDLC66 (0.1 MOI) for 1 h. Subsequently, these cells and cellular supernatants were meticulously collected to facilitate the evaluation of EqHV-8 replication through western blot and TCID_50_ analysis. Our findings revealed that Blebbistatin exerts a profound inhibitory influence on gD expression level and progeny virus titer in RK-13 cells, as elucidated in Figures 2A,B. Remarkably, Parallel observations were noted in MDBK cells (Figures 2C,D).

### 3.3 Antiviral activity of Blebbistatin against diverse EqHV-8 strains

**Figure 3:**
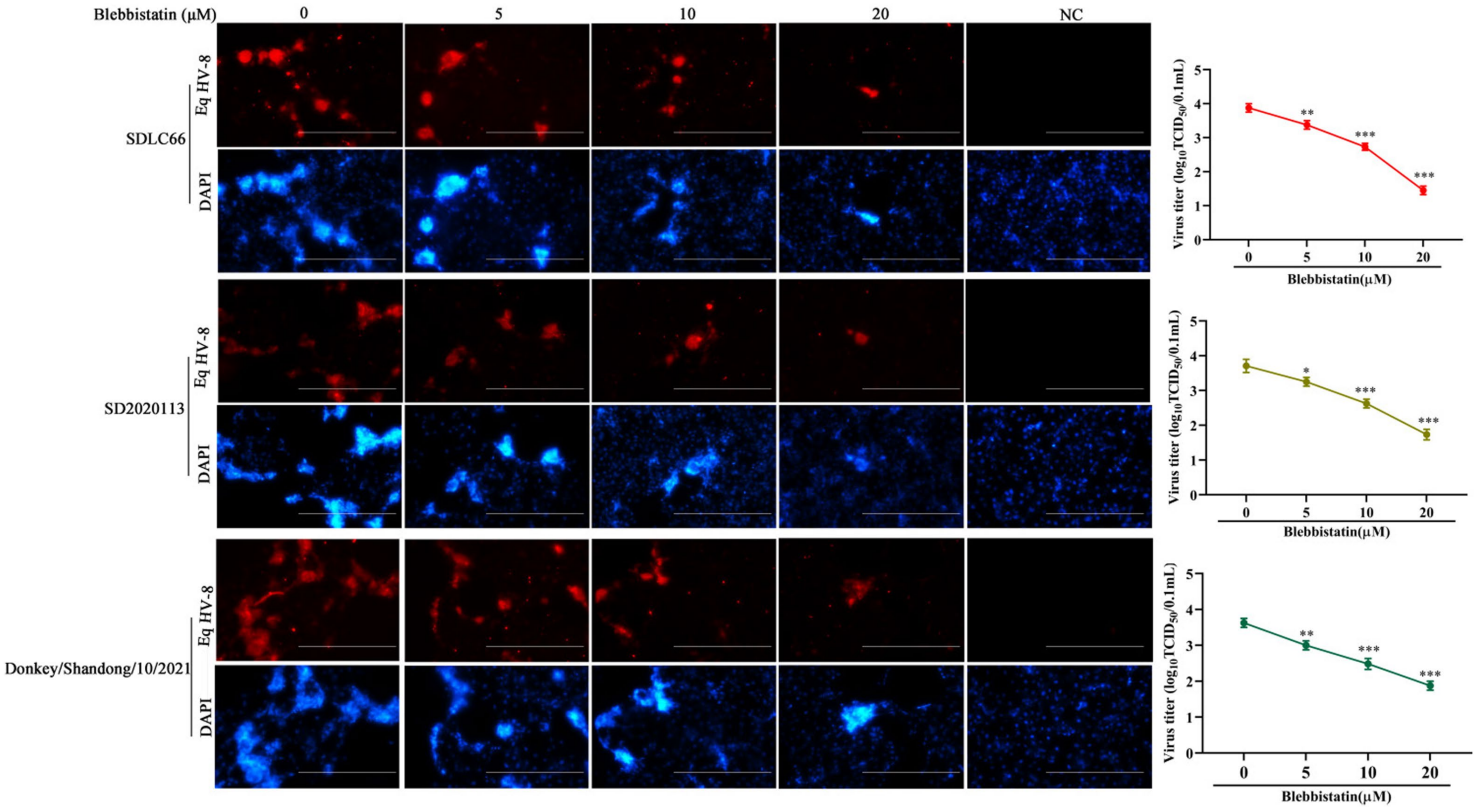
Blebbistatin show antiviral activity against other EqHV-8 strains. RK-13 cells were pretreated with Blebbistatin at different concentrations (0, 5, 10, or 20 μM) or DMSO for 1 h at 37°C, respectively, infected with EqHV-8 SDLC66, SD2020113, or Donkey/Shandong/10/2021 at 0.1MOI for 1 h in the presence of Blebbistatin at indicated concentrations. These cells were fixed with paraformaldehyde and stained using mouse anti-EHV-8 positive serum to detect EHV-8 proteins at 36 hpi (red), and the nucleocapsid was counterstained with DAPI (blue). Images were captured using Leica microsystems (DMi8, Germany). Scale bar, 100 μm. The mock-infected cells were served as negative control. Additionally, the progeny viral titer in cellular supernatants was analyzed by TCID_50_. Data were presented as the means of normalized data ± standard deviations (error bars) based on at least three independent experiments. *p < 0.05; **p < 0.01; ***p < 0.001.

In order to ascertain whether Blebbistatin exhibits antiviral properties against various strains of EqHV-8, a comprehensive investigation was conducted. RK-13 cells were pre-treated with Blebbistatin at 20 μM for 2 h, followed by their exposure to three distinct EqHV-8 strains, namely EqHV-8 SDLC66, EqHV-8 SD2020113, and EqHV-8 donkey/2021, each at 0.1 MOI for 1 h. Subsequently, the culture medium was replaced with 3% FBS MEM containing Blebbistatin, and these cells were fixed at 36 hpi to assess EqHV-8 replication via IFA. Meanwhile, those cellular supernatants were also collected to detect progeny virus titers by TCID_50_. Our results showed that Blebbistatin significantly reduces the infection efficiency of diverse EqHV-8 strains in RK-13 cells in a dose-dependent manner, as represented in Figure 3. These data indicated that Blebbistatin exerts a broad spectrum of inhibitory effects against different EqHV-8 strains.

### 3.4 Blebbistatin attenuates EqHV-8 infection during early stages

**Figure 4:**
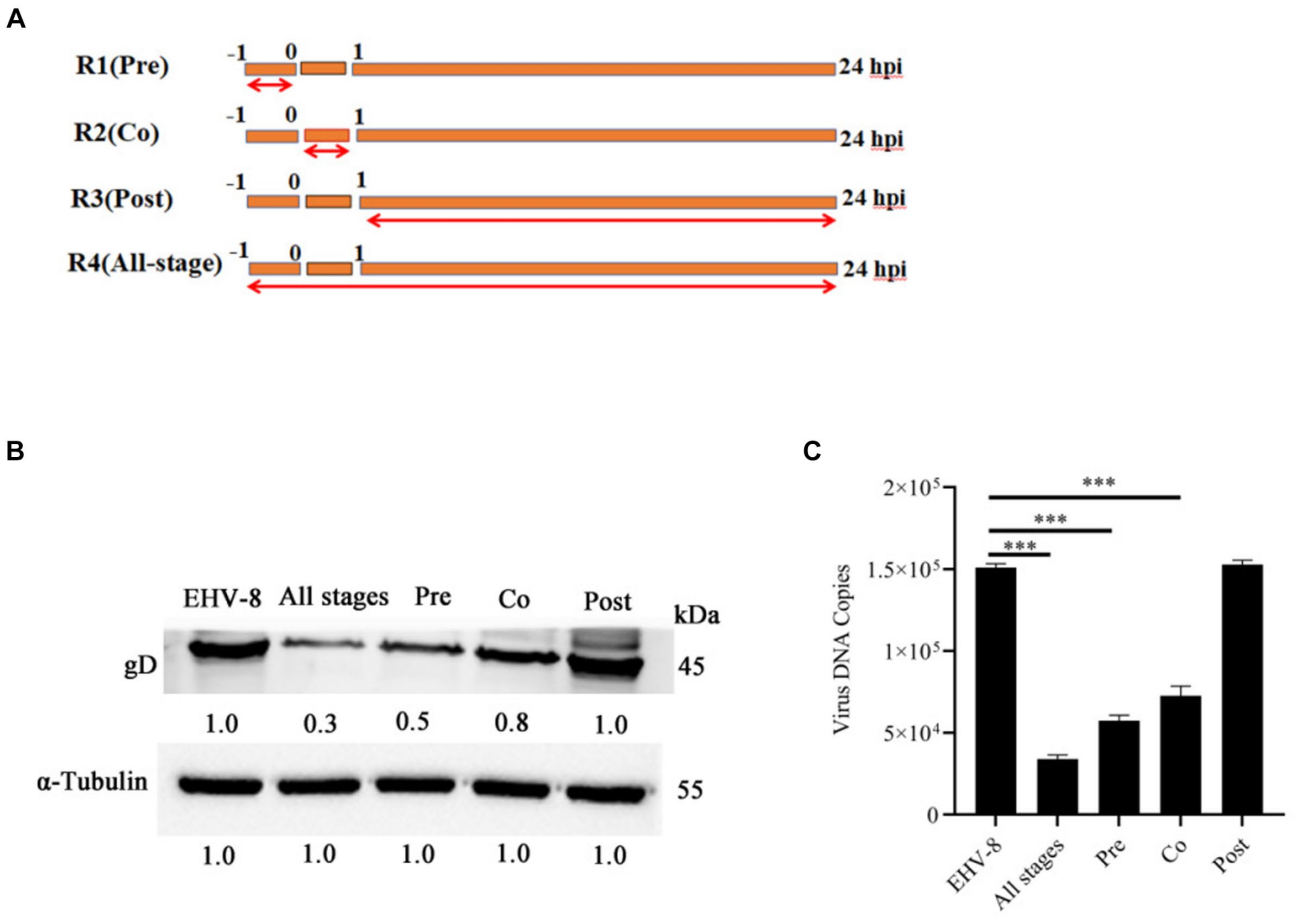
Blebbistatin inhibits EqHV-8 infection mainly at initial stage. Time-of-addition schematic **(A)**. The RK-13 cells were infected with EqHV-8 SDLC66 (MOI = 0.1) and treated with Blebbistatin at different time points, including before infection (Pre-treatment), during infection (Co-treatment), after infection (Post-treatment), and All-stage treatment. The RK-13 cells were treated with Blebbistatin (20 μM) as Pre-treatment, Co-treatment, Post-treatment and All-stage treatment for 24 hpi, and infected with EqHV-8 SDLC66 (MOI = 0.1), the cells were collected to detect gD expression for western blot **(B)**, meanwhile, the cell supernatants were also harvested to progeny virus generation by qPCR **(C)**. α-Tubulin acts as loading control, the data represent as mean ± SD. from three independent experiments, **p < 0.01; ***p < 0.001, ns: no significant.

To gain a deeper insight into the specific stage of the EqHV-8 life cycle that is perturbed by Blebbistatin, we performed a one-time course analysis experiment in RK-13 cells. As depicted in Figure 4A, our experiment was divided into four groups: R1, which represents the Blebbistatin pretreated group (Pre); R2, signifying the Blebbistatin and EqHV-8 co-treated group (Co); R3, representing the Blebbistatin post-treated group (Post); and R4, denoting the Blebbistatin treated group (All-stage). These cellular samples were harvested at 24 hpi to assess the gD expression. Our data demonstrate a significant reduction in gD protein expression in both R1 (Pre) and R2 (Co) groups when compared to the R3 (Post) group, as visually depicted in Figure 4B. This substantial decrease in gD expression suggests that Blebbistatin exerts an anti-EqHV-8 activity at an early stage of the viral life cycle. Furthermore, we examined the progeny virus production at 24 hpi by TCID_50_ assay. Our data reveals that a clear dose-dependent reduction in the copy number of EqHV-8 in both R1 (Pre) and R2 (Co) groups, as illustrated in Figure 4C. However, there were no visible changes observed in the R3 (Post) group.

### 3.5 Blebbistatin substantially reduces EqHV-8 entry into RK-13 cells

**Figure 5:**
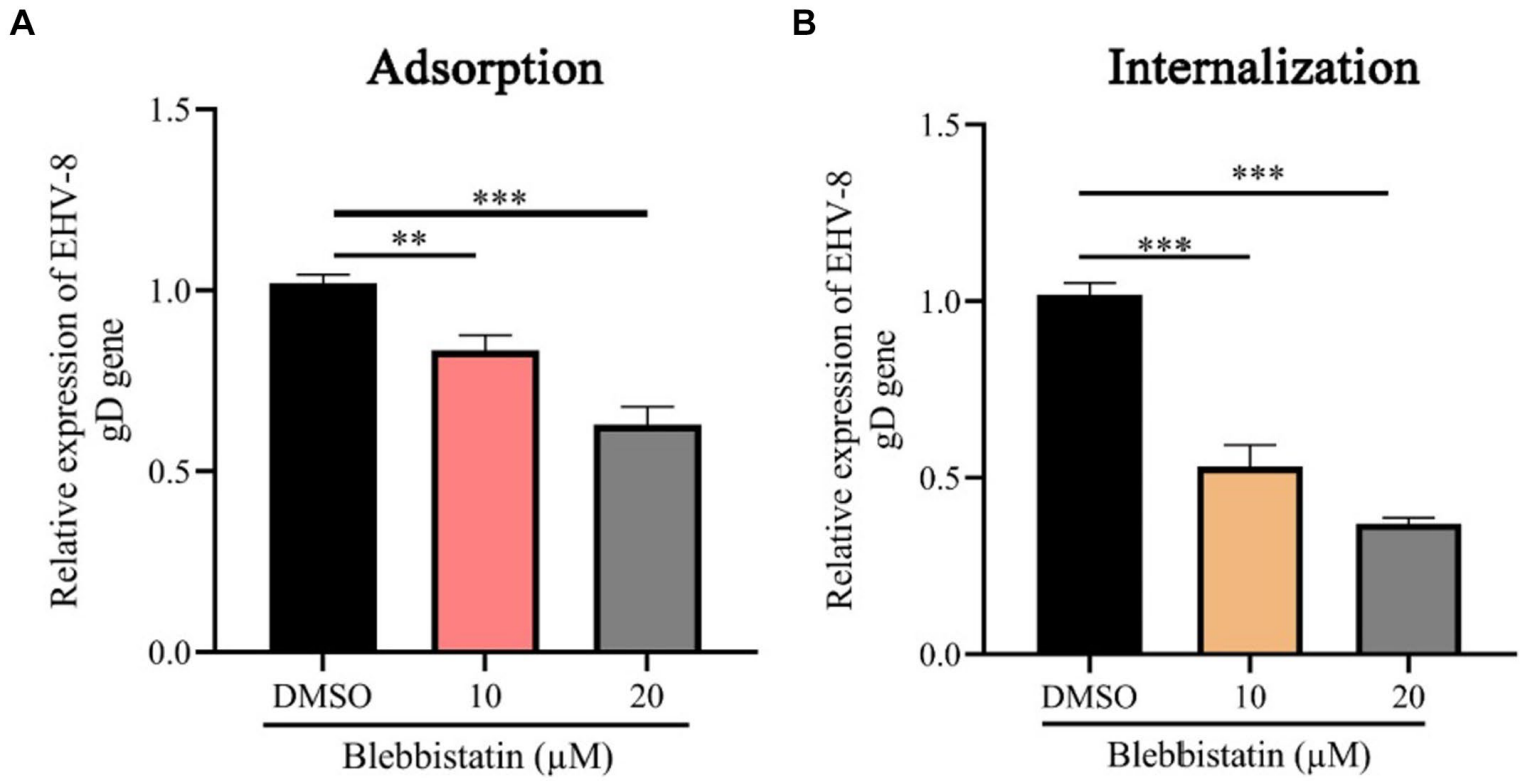
Blebbistatin suppress EqHV-8 replication at adsorption and internalization stage. The RK-13 cells were incubated with a mixture of Blebbistatin (20 μM) or DMSO and EqHV-8 (1 MOI) for 1 h at 4°C, and washed by PBS and incubated with 3% FBS MEM at 37°C for 24 h before the gD expression was tested using qPCR **(A)**. RK-13 cells were pretreated with Blebbistatin (20 μM) for 1 h and then incubated with EqHV-8 (MOI = 1) at 4°C for 1 h. The cells were washed with PBS and incubated with Blebbistatin (20 μM) or DMSO for another 1 h at 37°C, then washed by citrate buffer. The gD expression was detected using qPCR at 24 hpi. **(B)** **p < 0.01; ***p < 0.001; ns: not significant.

To investigate the impact of Blebbistatin treatment on the EqHV-8 binding and internalization processes within RK-13 cells, we conducted both virus adhesion and internalization assays. Our experimental findings yielded noteworthy results. At the adsorption stage, it was observed that RK-13 cells subjected to Blebbistatin treatment exhibited a notable reduction in gD expression in comparison to the group treated with DMSO (Figure 5A). This observation implies that Blebbistatin treatment significantly affects EqHV-8 infection during the initial adsorption phase. Similarly, during the internalization stage of EqHV-8 in RK-13 cells, we encountered analogous outcomes. The gD expression was notably decreased in RK-13 cells treated with Blebbistatin as compared to those treated with DMSO (Figure 5B). This provides further evidence that Blebbistatin exerts its influence on EqHV-8 infection not only at the adsorption stage but also during the internalization process.

### 3.6 Blebbistatin inhibits EqHV-8 infection *in vivo*

**Figure 6:**
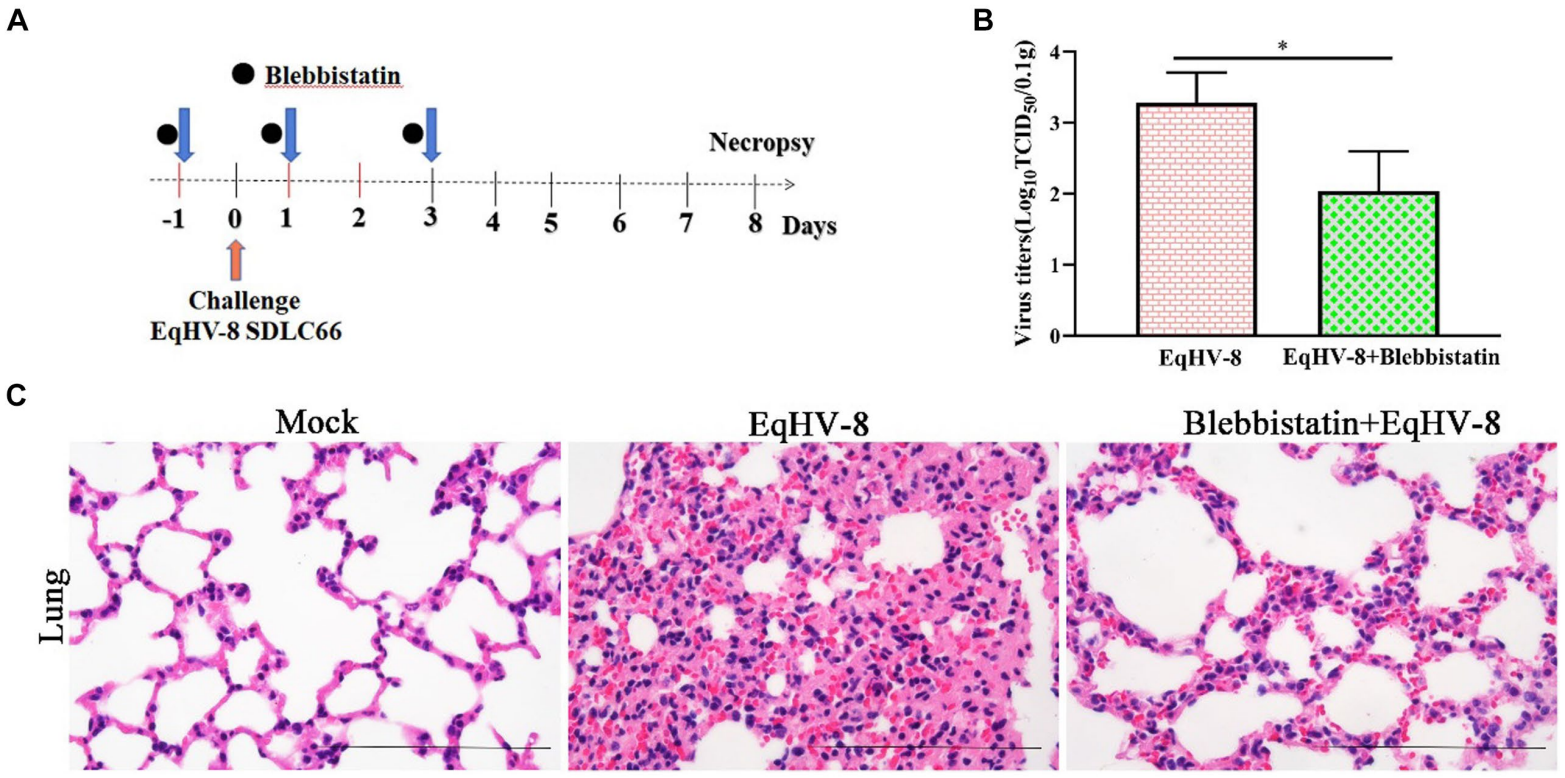
Blebbistatin inhibits EqHV-8 replication *in vivo* **(A)** The pattern diagram of the animal experiments. **(B)** The titration of EqHV-8 in lung from different group. RK-13 cells were used to titrate in lung from EqHV-8 infected or Blebbistatin treated EqHV-8 infected mice using the Reed–Muench method. The results are presented as the mean ± SD (error bars). *p < 0.05. **(C)** Representative images of hematoxylin and eosin (H&E) in the lungs derived from mice in indicated groups. Bar, 100 μm.

To assess the potential antiviral properties of Blebbistatin *in vivo*, we conducted an experiment involving BALB/C mice (Figure 6A). Our primary objective was to evaluate the extent of EqHV-8 replication within the lungs of these mice at 8 dpi. This assessment was achieved by titrating lung samples from different groups of mice on RK-13 cells, with the resulting data providing valuable insights into the antiviral effects of Blebbistatin. Our findings revealed a distinct contrast in viral replication between the groups. Specifically, the mean viral titers in the EqHV-8 group were notably elevated, measuring at 1.8 × 10^3^ TCID_50_, in contrast to the Blebbistatin+EqHV-8 group, where viral titers were substantially reduced, measuring at 1.1 × 10^2^ TCID_50_, as depicted in Figure 6B. These results underscore the significant inhibitory effect of Blebbistatin on EqHV-8 replication within the lungs of the mouse model. Moreover, histopathological examination of lung tissue specimens from the EqHV-8 group unveiled severe alveolar wall thickening, resulting in the compression and collapse of alveolar cavities, accompanied by a marked infiltration of inflammatory cells. In contrast, the lungs of BALB/C mice treated with Blebbistatin exhibited only minimal to mild alveolar wall thickening and inflammatory cell infiltration (Figure 6C). These histological observations further reinforce the notion that Blebbistatin exerts a substantial mitigating effect on EqHV-8-induced lung pathology in the mouse model. In addition, our findings strongly suggest that Blebbistatin could be considered a potential and promising antiviral drug candidate against EqHV-8 infection.

## 4 Discussion

In recent years, EqHV-8, a virus that affects donkeys in China, has caused significant economic losses in the donkey industry. Several cases of EqHV-8 infections in donkeys have led to issues like abortion, respiratory diseases, and viral encephalitis. Unfortunately, there are limited effective drugs against EqHV-8 infection. Our research has found that Blebbistatin can inhibit EqHV-8 in susceptible cells and under controlled *in vitro* conditions. This inhibition is due to Blebbistatin’s ability to modulate myosin II ATPase activity.

Blebbistatin is a compound known for inhibiting ATPase in myosin II, affecting processes like micropinocytosis and cellular blebbing (19–21). Recent studies show its potential in fighting viral infections. Recent research has unveiled Blebbistatin’s ability to suppress various viral infections. For instance, Veettil et al. demonstrated that Blebbistatin significantly reduces KSHV internalization into human dermal microvascular endothelial (HMVEC-d) cells (22). Similar antiviral effects have been reported against HSV-1, PRRSV, and MHV-68 in previous studies (13, 14, 16). In our experiments, Blebbistatin markedly decreased EqHV-8 infection in RK-13 and MDBK cells with dose-dependent properties (Figure 2). Additionally, our investigation delved into the intricate antiviral mechanisms underlying the action of Blebbistatin. Our findings revealed that Blebbistatin exerts its inhibitory influence on EqHV-8 replication during the early entry stage, as elucidated in Figure 4. Furthermore, our findings substantiate that Blebbistatin hampers EqHV-8 replication during the adsorption and internalization stages (Figure 5). Importantly, *in vivo* experiments confirmed the protective effect of Blebbistatin (30 μM/kg) against EqHV-8 in a mouse model, where it reduced viral replication and mitigated lung damage (Figure 6).

The involvement of myosins, a family of motor proteins with common features including ATP hydrolysis (ATPase enzymatic activity), kinetic energy transduction potential, and actin binding, is noteworthy in the context of viral infections (23). Previous studies have shown that nonmuscle myosin heavy chain IIA (NMHC-IIA) plays a crucial role in mediating HSV-1 entry, with Blebbistatin significantly reducing HSV-1 infection by inhibiting myosin II ATPase activity (24). Similar observations were made for PRRSV, where NMHC-IIA facilitates viral entry, and Blebbistatin notably reduces PRRSV replication via affecting myosin II ATPase activity (14, 15). In the current study, Blebbistatin significantly attenuated EqHV-8 replication in RK-13, MDBK cells, and in a mouse model (Figures 2, 6). Further research into the intricate mechanisms of Blebbistatin’s action and its interaction with non-muscle myosin heavy chain IIA is imperative for a comprehensive understanding of its antiviral properties. Understanding the role of myosin II and related host factors in EqHV-8 pathogenesis could pave the way for novel therapeutic strategies against EqHV-8 infection. Furthermore, future studies using primary equine cells or *in vivo* models are warranted to validate the findings. While the current study utilized an *in vivo* mouse model to assess the anti-EqHV-8 activity of Blebbistatin’s, it is important to note that mice may not fully elucidate the pathogenesis and immune response observed in horses and donkeys infected with EqHV-8. Therefore, additional research using equine-specific models is necessary to confirm the drug’s efficacy and safety. Altogether, our study not only reinforces the antiviral potential of Blebbistatin against EqHV-8 but also highlights the need for continued research into the mechanistic aspects of its action by utilizing equine-specific models.

## 5 Conclusion

In conclusion, our study provides compelling evidence of Blebbistatin’s significant inhibitory effect on EqHV-8 replication, demonstrated in both *in vitro* and *in vivo* experimental models. While our results highlight Blebbistatin’s potential as a novel antiviral agent against EqHV-8, further research using equine-specific models is warranted to validate its efficacy and safety in horses and donkeys.

## Data availability statement

The original contributions presented in the study are included in the article/Supplementary material, further inquiries can be directed to the corresponding authors.

## Ethics statement

The animal study was approved by all experimental protocols were approved by the Liaocheng University Animal Care and Use Committee (permit number: LC2023-11). The study was conducted in accordance with the local legislation and institutional requirements.

## Author contributions

LL: Data curation, Formal analysis, Investigation, Methodology, Resources, Software, Writing – original draft, Writing – review & editing. XC: Data curation, Investigation, Methodology, Software, Writing – original draft, Writing – review & editing. WL: Data curation, Software, Writing – review & editing. YL: Data curation, Methodology, Writing – review & editing. SL: Methodology, Software, Writing – review & editing. LC: Data curation, Software, Writing – review & editing. MK: Conceptualization, Supervision, Validation, Writing – original draft, Writing – review & editing. CW: Conceptualization, Funding acquisition, Project administration, Resources, Supervision, Visualization, Writing – original draft, Writing – review & editing. TW: Conceptualization, Data curation, Funding acquisition, Investigation, Methodology, Project administration, Resources, Software, Supervision, Validation, Writing – original draft, Writing – review & editing. YY: Methodology, Formal analysis, Validation, Writing – review & editing. QS: Methodology, Formal analysis, Validation, Writing – review & editing.
