# Supplementary material for: Blebbistatin as a novel antiviral agent targeting equid herpesvirus type 8
Source: Front Vet Sci. 2024 Jun 5;11:1390304. doi: 10.3389/fvets.2024.1390304 (PMC11186319; doi:10.3389/fvets.2024.1390304)
Supplement: Supplementary file 1 [file Table_1.DOCX]

Supplementary Table 1.The primers in this study.

| Primers | Primer sequences（5´-3´） |
| --- | --- |
| ORF72-F | CCCACGTGTGCAACGCCTAT |
| ORF72-R | ATACAGTCCCGAGGCAGAGT |
| GAPDH-F | CCTTCCGTGTCCCTACTGCCAAC |
| GAPDH-R | GACGCCTGCTTCACCACCTTCT |
