# Supplementary figures and images for: Blebbistatin as a novel antiviral agent targeting equid herpesvirus type 8
Source: Front Vet Sci. 2024 Jun 5;11:1390304. doi: 10.3389/fvets.2024.1390304 (PMC11186319; doi:10.3389/fvets.2024.1390304)

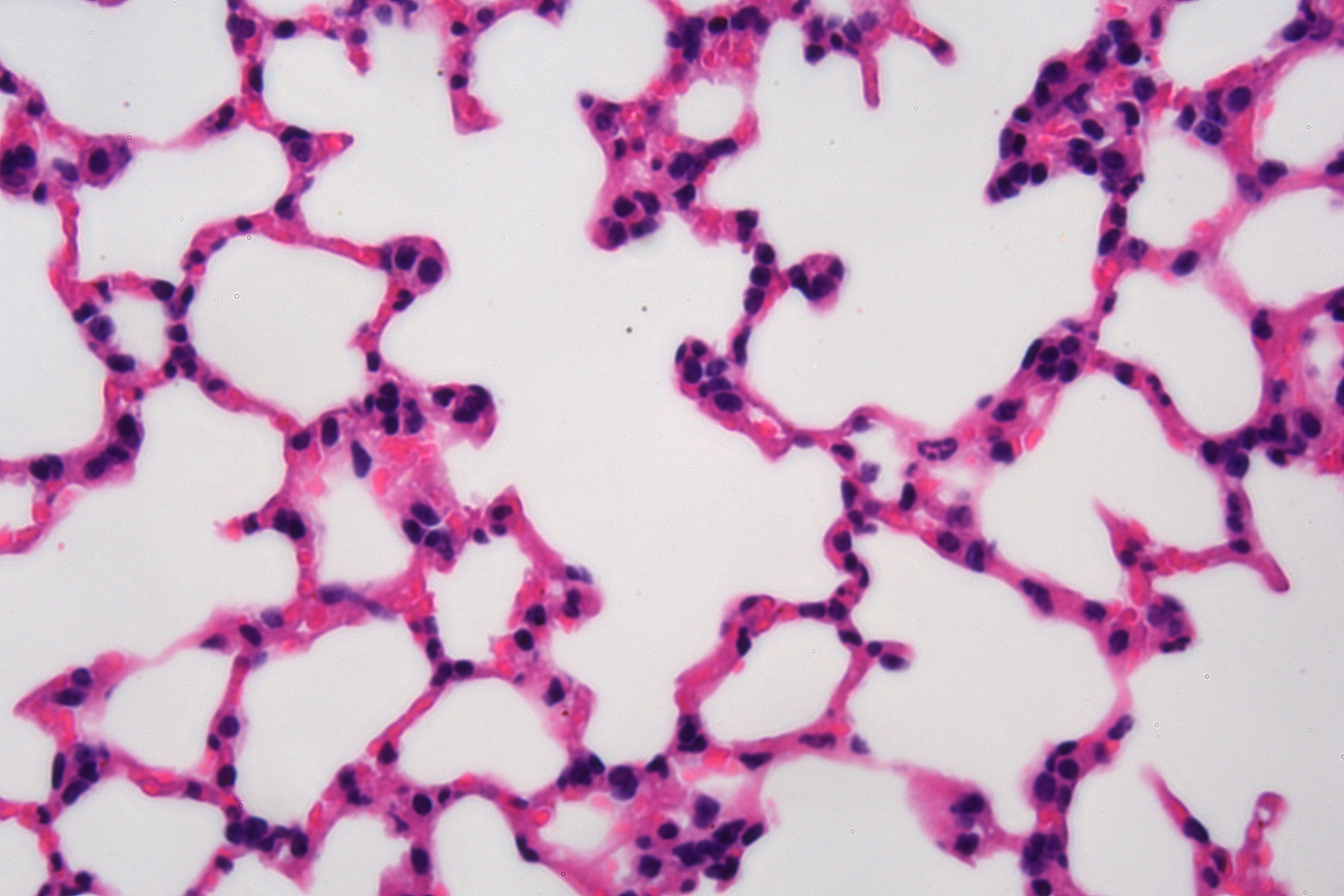

Supplement: Supplementary file 2 [file Data_Sheet_1.ZIP › 01-microscope figure-BBT-Blebbistatin-1390304- Raw data-2024/Mock.png]

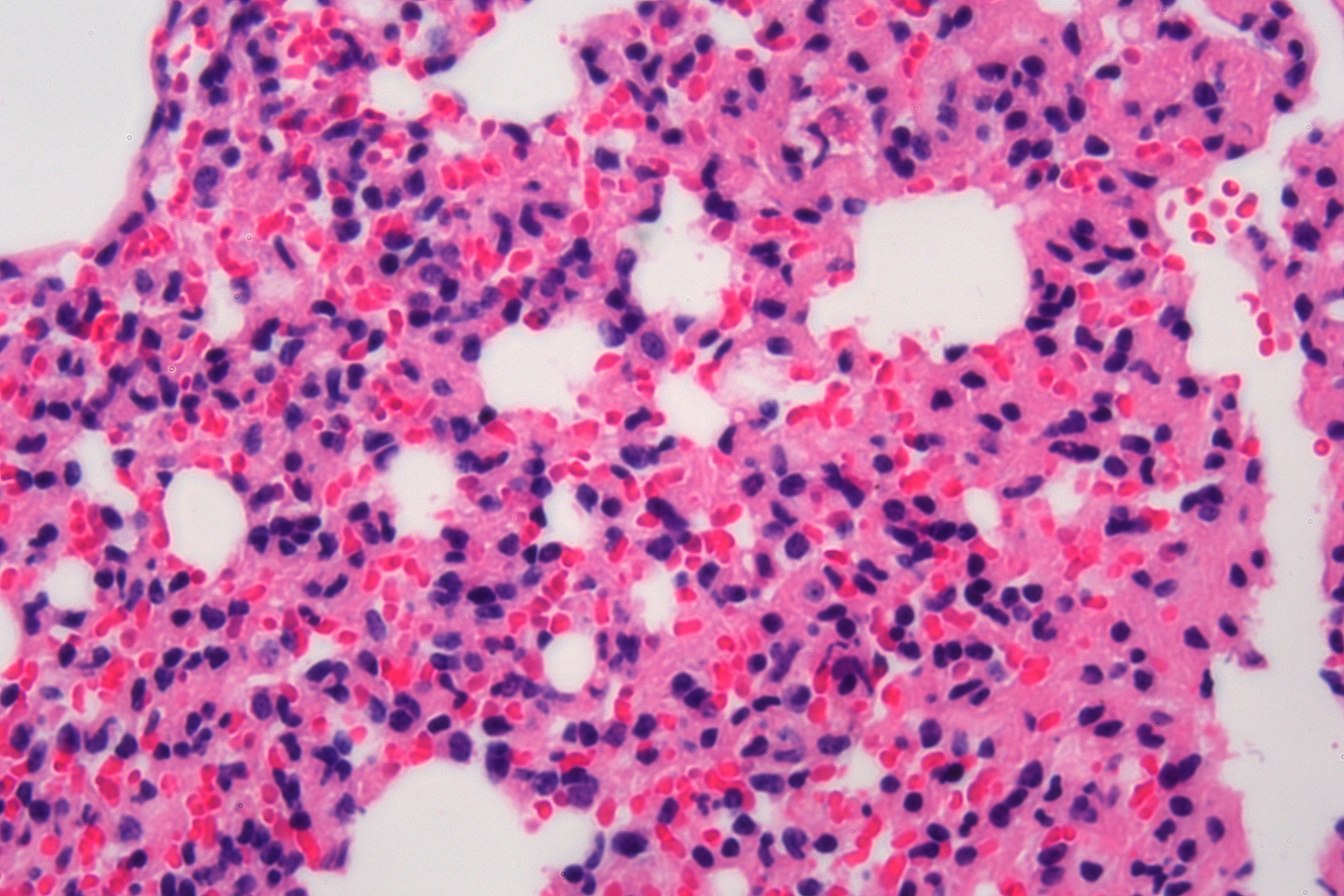

Supplement: Supplementary file 3 [file Data_Sheet_2.ZIP › microscope figure-BBT-Blebbistatin-1390304- Raw data-2024/EqHV-8.png]

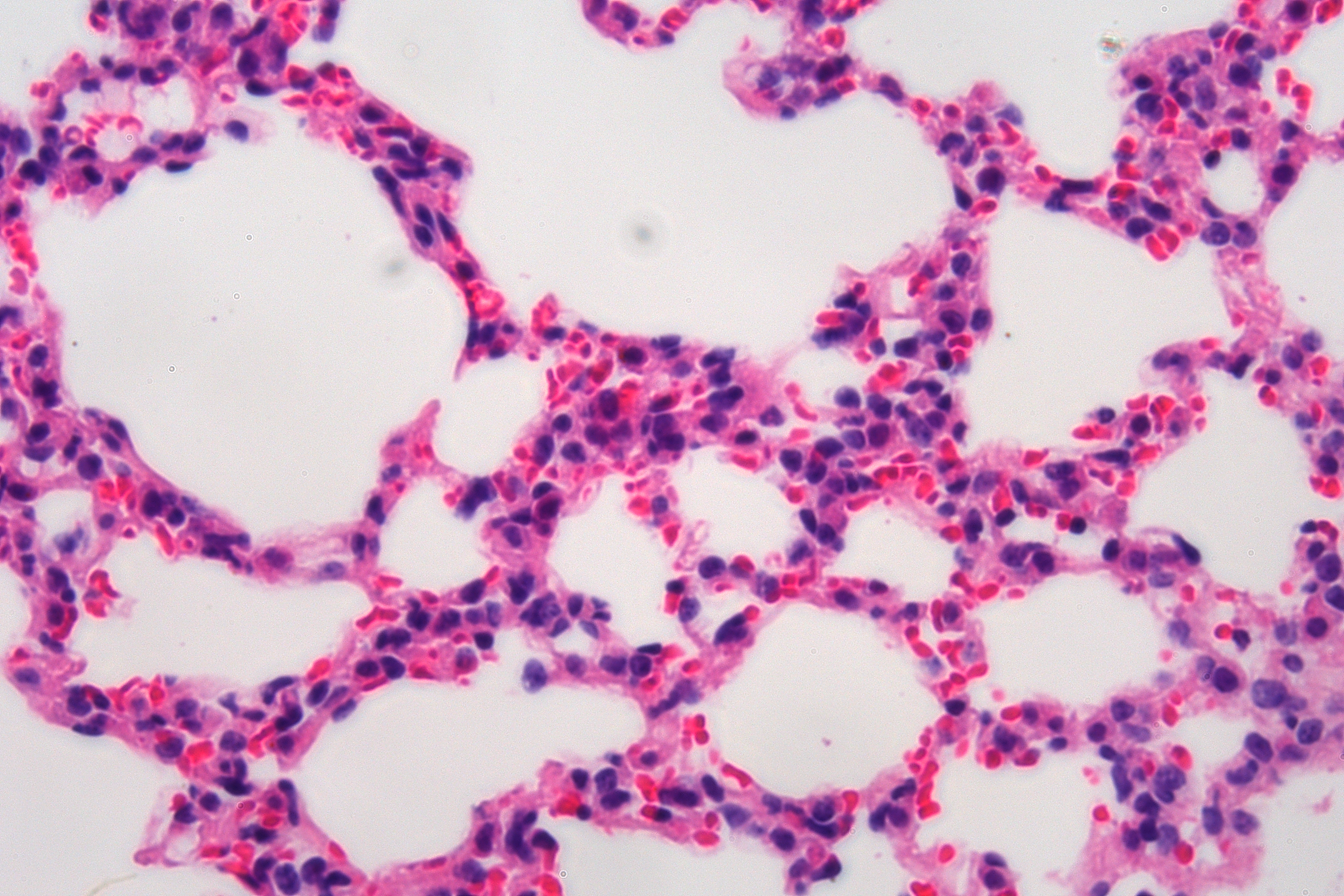

Supplement: Supplementary file 3 [file Data_Sheet_2.ZIP › microscope figure-BBT-Blebbistatin-1390304- Raw data-2024/EqHV-8+BBT.png]
