# Supplementary material for: Blebbistatin as a novel antiviral agent targeting equid herpesvirus type 8
Source: Front Vet Sci. 2024 Jun 5;11:1390304. doi: 10.3389/fvets.2024.1390304 (PMC11186319; doi:10.3389/fvets.2024.1390304)
Supplement: Supplementary file 4 [file Data_Sheet_3.ZIP › Labelled Original Figures 2024(2) BBT.docx]

All original western blot figures, including intensity ratio of each band, were shown in below, and the blot membrane were cropped to incubate different antibodies, so there are not all molecular weight markers.


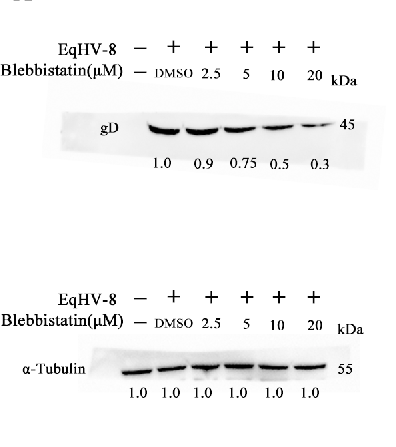


**Fig. 2B** RK-13 cells were pre-incubated with Blebbistatin at different concentrations for 1 h and then infected with EqHV-8 SDLC66 at 0.1 MOI. The gD expression was analyzed by western blot (B)


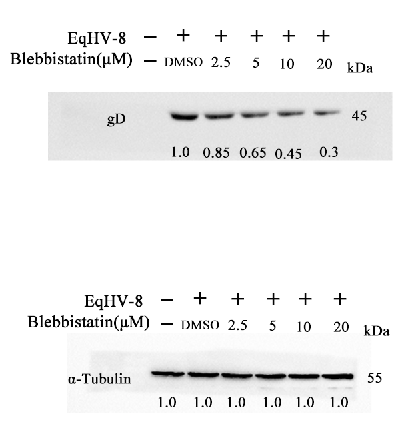


**Fig. 2D** MDBK cells were pre-incubated with different concentrations of Blebbistatin for 1 h and afterward infected with EqHV-8 SDLC66 at 0.1 MOI. The gD protein expression was analyzed by western blotting (2D).


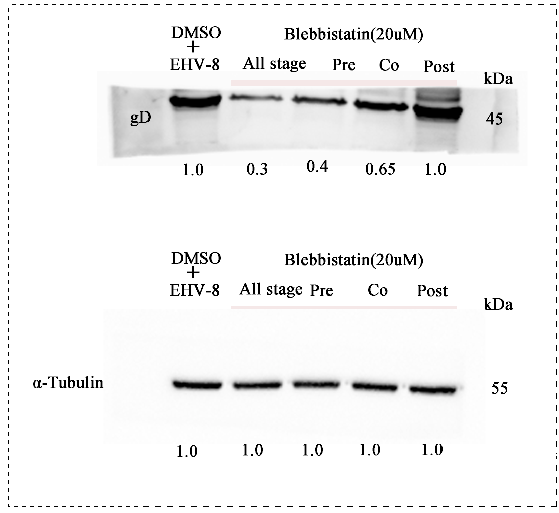

**Fig. 3B** The RK-13 cells were infected with EqHV-8 SDLC66 (MOI=0.1) and treated with Blebbistatin (20μM) at different times of infection, including All-stage treatment, Pre-treatment, Co-treatment, and Post-treatment. The expression of gD protein expression was determined by western blot.
